# 4-{2-[4-(Dimethyl­amino)phen­yl]ethyl­idene}benzonitrile

**DOI:** 10.1107/S1600536809018674

**Published:** 2009-05-23

**Authors:** Rodolfo Moreno-Fuquen, Richard Dvries, Jahyr Theodoro, Javier Ellena

**Affiliations:** a Departamento de Química, Facultad de Ciencias, Universidad del Valle, Apartado 25360, Santiago de Cali, Colombia; b Instituto de Física de São Carlos, Universidade de São Paulo, USP, São Carlos, SP, Brazil

## Abstract

In the crystal of the title compound, C_17_H_16_N_2_, mol­ecules are linked by C—H⋯N hydrogen bonds, forming rings of graph-set motifs *R*_2_^1^(6) and *R*_2_^2^(10). The title mol­ecule is close to planar, with a dihedral angle between the aromatic rings of 0.6 (1)°. Torsion angles confirm a conformational *trans* structure.

## Related literature

For background information on photonic materials, see: Blanchard-Desce *et al.* (1988 ▶); Lapouyade *et al.* (1993 ▶); Papper *et al.* (1997 ▶). For background information on spectroscopic properties, see: Daum *et al.* (1995 ▶); Kubicki (2007 ▶). For graph-set motifs, see: Etter (1990 ▶). For related literature, see: Craig *et al.* (2006 ▶); Maryanoff & Reitz (1989 ▶).
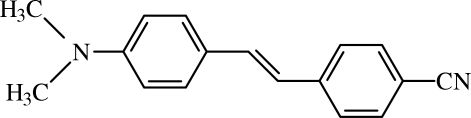


## Experimental

### 

#### Crystal data

C_17_H_16_N_2_
*M* *_r_* = 248.32
Monoclinic, 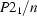

*a* = 6.2009 (2) Å
*b* = 7.9706 (3) Å
*c* = 27.9619 (11) Å
β = 93.6027 (13)°
*V* = 1379.28 (9) Å^3^
*Z* = 4
Mo *K*α radiation
μ = 0.07 mm^−1^
*T* = 291 K
0.28 × 0.14 × 0.08 mm

#### Data collection

Bruker–Nonius KappaCCD diffractometer
Absorption correction: none
4640 measured reflections
2443 independent reflections
1428 reflections with *I* > 2σ(*I*)
*R*_int_ = 0.037

#### Refinement

*R*[*F* ^2^ > 2σ(*F* ^2^)] = 0.053
*wR*(*F* ^2^) = 0.173
*S* = 1.02
2443 reflections
192 parameters
H-atom parameters constrained
Δρ_max_ = 0.12 e Å^−3^
Δρ_min_ = −0.16 e Å^−3^

### 

Data collection: *COLLECT* (Nonius, 2000 ▶); cell refinement: *SCALEPACK* (Otwinowski & Minor, 1997 ▶); data reduction: *DENZO* (Otwinowski & Minor, 1997 ▶) and *SCALEPACK*; program(s) used to solve structure: *SHELXS97* (Sheldrick, 2008 ▶); program(s) used to refine structure: *SHELXL97* (Sheldrick, 2008 ▶); molecular graphics: *ORTEP-3 for Windows* (Farrugia, 1997 ▶); software used to prepare material for publication: *PARST95* (Nardelli, 1995 ▶).

## Supplementary Material

Crystal structure: contains datablocks I, global. DOI: 10.1107/S1600536809018674/bv2116sup1.cif

Structure factors: contains datablocks I. DOI: 10.1107/S1600536809018674/bv2116Isup2.hkl

Additional supplementary materials:  crystallographic information; 3D view; checkCIF report

## Figures and Tables

**Table 1:** Hydrogen-bond geometry (Å, °)

*D*—H⋯*A*	*D*—H	H⋯*A*	*D*⋯*A*	*D*—H⋯*A*
C1—H1*C*⋯N2^i^	0.96	2.99	3.858 (3)	151
C2—H2*C*⋯N2^i^	0.96	2.80	3.646 (3)	147
C13—H13⋯N2^ii^	0.93	2.88	3.696 (3)	147

Symmetry codes: (i) 
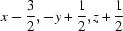
; (ii) 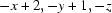.

**Fig. 1.**
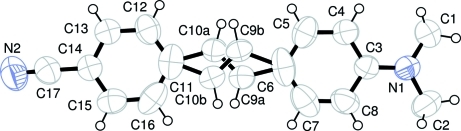
An ORTEP-3 (Farrugia, 1997) plot of the DCS compound, with the atomic labelling scheme. The shapes of the ellipsoids correspond to 50% probability contours of atomic displacement and, for the sake of clarity, H atoms are shown as spheres of arbitrary radius.

**Fig. 2.**
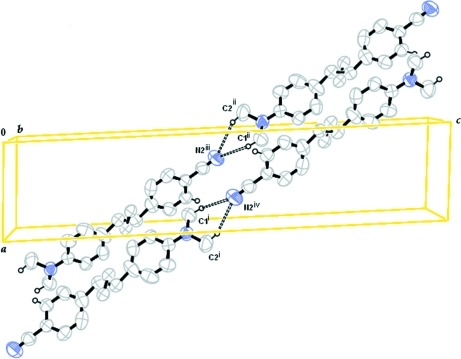
(Supp. Mat). Part of the crystal structure of DCS showing the formation of R21(6) rings and generating sheets which lie in the (104) plane. [Symmetry codes: (i) 1 + x, y, z; (ii) -x, 1/2 + y, 1 - z; (iii) 3/2 - x,1/2 + y, 1/2 - z; (iv) -1/2 + x, 1/2 - y, 1/2 + z.]

**Fig. 3.**
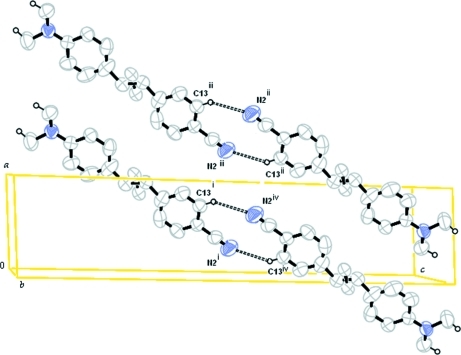
(Supp. Mat). Part of the crystal structure of DCS showing the formation of R22(10) rings which are running parallel to the [112] direction. [Symmetry codes: (i) 3/2 - x, -1/2 + y, 1/2 - z; (ii) 1/2 + x, 1/2 - y, 1/2 + z; (iii) 5/2 - x, -1/2 + y, 1/2 - z; (iv) -1/2 + x, 1/2 - y, 1/2 + z.]

## supplementary crystallographic information

### Comment

Polyene systems are often used as π conjugating units as they provide an effective pathway for the efficient push-pull charge transfer between the donor and acceptor groups (Blanchard-Desce *et al.*, 1988). Stilbene materials are expected to have diverse applications in photochemistry, fluorescense and non linear optical (NLO) processes when donor-acceptor substituents are introduced in the phenyl rings in order to spread the conjugation over the whole molecule (Lapouyade *et al.*, 1993; Papper *et al.*, 1997). Spectroscopic properties of the title 4-dimethylamino-4-cyano-stilbene (DCS) system have been extensively studied (Daum *et al.*, 1995; Kubicki, 2007). Nevertheless, a survey of the literature shows that crystallographic information of stilbene compounds is still rather scarce.

The main aim of this work is to present the molecular and crystal structure of the DCS, one of the best exponents of the stilbene series, to analyse its configuration, its C=C double bond and to show the supramolecular arranging of the system. A perspective view of the molecule of the title compound, showing the atomic numbering scheme, is given in Fig. 1. The DCS molecule suffers a rotational disorder, atoms C9 and C10 were modeled as exchanged with a minor occupancy fraction refined to 39 (1)%. The dimethylamino group forms a dihedral angle of 20.9 (2)° with respect to its phenyl ring. The phenyl rings of the title structure is almost coplanar showing a dihedral angle of 0.6 (1)° between the planes of the rings. The phenyl rings are twisted out of the ethylene bond plane, and are defined by the torsion angles C16—C11=C10A—C9A and C10A=C9A—C6—C5 (Table 1). The C9A=C10A bond length is close to the reported value for the ethylene C=C bond length [1.330 (1) A°] (Craig *et al.*, 2006). The title molecule shows a torsion angle C6 C9A C10A C11 equal to 177.4 (3)° and it also shows that the bond angles between the olefinic double bond and the two aromatic rings C6—C9A=C10A and C11—C10A=C9A are close to 120° (Table 1) indicating small repulsion between the aromatic rings. These values allow to define its configuration as *trans*.

The molecules of the title compound are linked into sheets by a C—H···N weak intermolecular interactions (Table 2) (Nardelli, 1995) generating two dimensional substructures. In the first substructure, methyl amino C1 and C2 atoms in the molecule at (1 + *x*, *y*, *z*) acts as a hydrogen bond donors to cyano N2 atom in the molecule at (-1/2 + *x*, 1/2 - *y*, 1/2 + *z*) so forming a *R*_2_^1^(6) rings (Etter, 1990) and generating sheets which lie in the (104) plane (Fig. 2, Supp. Mat). In the second substructure, atom C13 in the molecule at (*x*, *y*, *z*) acts as a hydrogen bond donor to cyano N2 atom in the molecule at (3/2 - *x*, -1/2 + *y*, 1/2 - *z*) so forming a *R*_2_^2^(10) rings which are running parallel to the [112] direction (Fig. 3, Supp. Mat).

### Experimental

By means of Wittig reaction (Maryanoff & Reitz, 1989), the 4-dimethylamino-benzyl-triphenylphosphonium iodide was prepared. The title stilbene was obtained by the reaction of equimolar quantities of phosphonium salt and 4-cyano benzaldehyde (0.02 mol) in THF solution. The mixture was maintained with stirring under argon atmosphere. The reaction mixture was kept at 273 K and it was dropped with a solution of *tert*-butanol and potassium *tert*-butoxide. Crystals of suitable quality for single-crystal X-ray diffraction were grown in chloroform. Thin layer chromatography (TLC) was used to confirm the structure of the individual compounds. IR spectra were recorded on a Shimadzu FT—IR 8400 spectrophotometer.

*N*-(*p*-chlorophenyl)maleimide. Yellow crystals; yield 40%; mp 384 (1) K. IR (KBr) 3051 cm^-1^ (C—H), 2922 cm^-1^ (=C—H), 2217 cm^-1^ (C—N), 1590 cm^-1^ (C=C).

### Refinement

The space group P 2_1_/n was uniquely assigned from the systematic absences. All H-atoms were located from difference maps and then they were treated as riding atoms [C_aro_—H= 0.93 A° and C_sp3_—H= 0.96 A°, *U*_iso_(H)= 1.2*U*_eq_(C_aro_), *U*_iso_(H)= 1.5*U*_eq_(C_sp3_)).

Some reflections were omitted in the FCF file perhaps because the crystal was placed in a position very close to the detector and therefore some diffracted reflections at low angle were potentially covered by the beamstop.

### Crystal data

**Table:**

C_17_H_16_N_2_	*F*(000) = 528
*M**_r_* = 248.32	*D*_x_ = 1.196 Mg m^−^^3^
Monoclinic, *P*2_1_/*n*	Melting point: 384(1) K
Hall symbol: -P 2yn	Mo *K*α radiation, λ = 0.71073 Å
*a* = 6.2009 (2) Å	Cell parameters from 4640 reflections
*b* = 7.9706 (3) Å	θ = 2.9–27.5°
*c* = 27.9619 (11) Å	µ = 0.07 mm^−^^1^
β = 93.6027 (13)°	*T* = 291 K
*V* = 1379.28 (9) Å^3^	Prism, yellow
*Z* = 4	0.28 × 0.14 × 0.08 mm

### Data collection

**Table:**

Bruker–Nonius KappaCCD diffractometer	1428 reflections with *I* > 2σ(*I*)
Radiation source: fine-focus sealed tube	*R*_int_ = 0.037
horizonally mounted graphite crystal	θ_max_ = 25.1°, θ_min_ = 3.3°
Detector resolution: 9 pixels mm^-1^	*h* = −7→7
CCD scans	*k* = −9→9
4640 measured reflections	*l* = −33→33
2443 independent reflections	

### Refinement

**Table:**

Refinement on *F*^2^	Primary atom site location: structure-invariant direct methods
Least-squares matrix: full	Secondary atom site location: difference Fourier map
*R*[*F*^2^ > 2σ(*F*^2^)] = 0.053	Hydrogen site location: inferred from neighbouring sites
*wR*(*F*^2^) = 0.173	H-atom parameters constrained
*S* = 1.02	*w* = 1/[σ^2^(*F*_o_^2^) + (0.0926*P*)^2^ + 0.0631*P*] where *P* = (*F*_o_^2^ + 2*F*_c_^2^)/3
2443 reflections	(Δ/σ)_max_ < 0.001
192 parameters	Δρ_max_ = 0.12 e Å^−^^3^
0 restraints	Δρ_min_ = −0.16 e Å^−^^3^

### Special details

**Table:**

Geometry. All e.s.d.'s (except the e.s.d. in the dihedral angle between two l.s. planes) are estimated using the full covariance matrix. The cell e.s.d.'s are taken into account individually in the estimation of e.s.d.'s in distances, angles and torsion angles; correlations between e.s.d.'s in cell parameters are only used when they are defined by crystal symmetry. An approximate (isotropic) treatment of cell e.s.d.'s is used for estimating e.s.d.'s involving l.s. planes.
Refinement. Refinement of *F*^2^ against ALL reflections. The weighted *R*-factor *wR* and goodness of fit *S* are based on *F*^2^, conventional *R*-factors *R* are based on *F*, with *F* set to zero for negative *F*^2^. The threshold expression of *F*^2^ > σ(*F*^2^) is used only for calculating *R*-factors(gt) *etc*. and is not relevant to the choice of reflections for refinement. *R*-factors based on *F*^2^ are statistically about twice as large as those based on *F*, and *R*- factors based on ALL data will be even larger.

### Fractional atomic coordinates and isotropic or equivalent isotropic displacement parameters (Å^2^)

**Table:**

	*x*	*y*	*z*	*U*_iso_*/*U*_eq_	Occ. (<1)
N1	0.0557 (2)	0.34596 (18)	0.41210 (5)	0.0821 (5)	
N2	1.1813 (3)	0.3178 (3)	0.01870 (7)	0.1246 (8)	
C1	−0.1199 (3)	0.4594 (3)	0.41729 (7)	0.1036 (7)	
H1A	−0.2208	0.4503	0.3899	0.155*	
H1B	−0.0658	0.5721	0.4197	0.155*	
H1C	−0.1912	0.4320	0.4458	0.155*	
C2	0.1629 (4)	0.2885 (3)	0.45672 (7)	0.1152 (8)	
H2A	0.2872	0.3573	0.4645	0.173*	
H2B	0.2075	0.1740	0.4533	0.173*	
H2C	0.0648	0.2961	0.4819	0.173*	
C3	0.1650 (3)	0.34508 (19)	0.37074 (6)	0.0701 (5)	
C4	0.0836 (3)	0.4231 (2)	0.32872 (6)	0.0797 (5)	
H4	−0.0494	0.4772	0.3282	0.096*	
C5	0.1975 (4)	0.4211 (2)	0.28796 (7)	0.0934 (6)	
H5	0.1395	0.4750	0.2606	0.112*	
C6	0.3973 (4)	0.3406 (3)	0.28637 (9)	0.1056 (8)	
C7	0.4692 (4)	0.2609 (3)	0.32802 (11)	0.1121 (8)	
H7	0.5992	0.2026	0.3283	0.135*	
C8	0.3615 (3)	0.2627 (3)	0.36853 (8)	0.0935 (6)	
H8	0.4204	0.2074	0.3956	0.112*	
C9A	0.5574 (9)	0.3187 (5)	0.25116 (12)	0.0751 (9)	0.60
H9A	0.6791	0.2535	0.2588	0.075*	0.60
C10A	0.5323 (10)	0.3906 (6)	0.20808 (14)	0.0727 (9)	0.60
H10A	0.4141	0.4596	0.2005	0.073*	0.60
C11	0.6991 (4)	0.3586 (3)	0.17129 (8)	0.0981 (7)	
C12	0.6354 (3)	0.4322 (3)	0.12852 (8)	0.0905 (6)	
H12	0.5050	0.4902	0.1261	0.109*	
C13	0.7552 (3)	0.4241 (2)	0.08934 (6)	0.0803 (5)	
H13	0.7071	0.4769	0.0610	0.096*	
C14	0.9488 (3)	0.3369 (2)	0.09185 (6)	0.0732 (5)	
C15	1.0198 (3)	0.2606 (2)	0.13431 (7)	0.0889 (6)	
H15	1.1502	0.2025	0.1365	0.107*	
C16	0.8952 (5)	0.2714 (3)	0.17349 (7)	0.1034 (7)	
H16	0.9429	0.2194	0.2020	0.124*	
C17	1.0761 (3)	0.3277 (3)	0.05086 (7)	0.0880 (6)	
C9B	0.4576 (10)	0.3811 (9)	0.2327 (3)	0.0708 (13)	0.40
H9B	0.3677	0.4425	0.2113	0.071*	0.40
C10B	0.6456 (9)	0.3229 (8)	0.2215 (3)	0.0737 (13)	0.40
H10B	0.7370	0.2629	0.2429	0.074*	0.40

### Atomic displacement parameters (Å^2^)

**Table:**

	*U*^11^	*U*^22^	*U*^33^	*U*^12^	*U*^13^	*U*^23^
N1	0.0877 (10)	0.0838 (10)	0.0747 (10)	0.0108 (8)	0.0039 (8)	0.0070 (7)
N2	0.1098 (15)	0.150 (2)	0.1173 (16)	0.0013 (13)	0.0338 (13)	−0.0152 (13)
C1	0.0975 (14)	0.1195 (17)	0.0957 (14)	0.0204 (13)	0.0203 (11)	0.0006 (12)
C2	0.1341 (19)	0.1260 (18)	0.0844 (14)	0.0219 (15)	−0.0015 (13)	0.0214 (12)
C3	0.0744 (11)	0.0586 (10)	0.0770 (11)	−0.0002 (8)	0.0043 (9)	−0.0007 (8)
C4	0.0851 (12)	0.0759 (12)	0.0776 (12)	0.0027 (10)	0.0001 (9)	−0.0036 (9)
C5	0.1241 (17)	0.0847 (13)	0.0718 (12)	−0.0215 (13)	0.0105 (11)	−0.0066 (10)
C6	0.1192 (19)	0.0875 (15)	0.1158 (18)	−0.0355 (14)	0.0531 (16)	−0.0418 (13)
C7	0.1003 (17)	0.0895 (16)	0.150 (2)	0.0056 (13)	0.0389 (17)	−0.0172 (16)
C8	0.0871 (13)	0.0776 (13)	0.1165 (16)	0.0126 (11)	0.0113 (12)	0.0065 (11)
C9A	0.077 (2)	0.071 (2)	0.076 (2)	0.0044 (19)	−0.003 (2)	−0.0016 (19)
C10A	0.072 (3)	0.068 (2)	0.077 (3)	0.001 (2)	0.000 (2)	−0.002 (2)
C11	0.1216 (18)	0.0869 (15)	0.0880 (15)	−0.0386 (14)	0.0244 (14)	−0.0234 (12)
C12	0.0883 (13)	0.0880 (14)	0.0967 (15)	−0.0082 (10)	0.0177 (11)	−0.0184 (11)
C13	0.0801 (12)	0.0784 (12)	0.0821 (12)	−0.0027 (9)	0.0024 (10)	0.0007 (9)
C14	0.0737 (11)	0.0689 (11)	0.0769 (12)	−0.0083 (9)	0.0034 (9)	−0.0078 (9)
C15	0.0949 (14)	0.0773 (12)	0.0920 (14)	0.0007 (11)	−0.0143 (11)	−0.0034 (10)
C16	0.153 (2)	0.0870 (14)	0.0688 (13)	−0.0326 (15)	−0.0075 (13)	0.0035 (10)
C17	0.0785 (12)	0.0918 (14)	0.0936 (14)	−0.0045 (11)	0.0064 (11)	−0.0123 (11)
C9B	0.072 (4)	0.066 (3)	0.072 (4)	−0.003 (3)	−0.010 (3)	0.002 (3)
C10B	0.078 (3)	0.072 (3)	0.070 (4)	0.002 (3)	−0.005 (3)	0.005 (3)

### Geometric parameters (Å, °)

**Table:**

N1—C3	1.377 (2)	C9A—C10A	1.334 (6)
N1—C1	1.430 (2)	C9A—H9A	0.9300
N1—C2	1.451 (2)	C9A—H10B	1.2345
N2—C17	1.146 (2)	C10A—C11	1.525 (6)
C1—H1A	0.9600	C10A—H10A	0.9300
C1—H1B	0.9600	C10A—H9B	1.1103
C1—H1C	0.9600	C11—C12	1.368 (3)
C2—H2A	0.9600	C11—C16	1.399 (3)
C2—H2B	0.9600	C11—C10B	1.491 (8)
C2—H2C	0.9600	C12—C13	1.363 (2)
C3—C8	1.389 (3)	C12—H12	0.9300
C3—C4	1.396 (2)	C13—C14	1.385 (2)
C4—C5	1.378 (3)	C13—H13	0.9300
C4—H4	0.9300	C14—C15	1.381 (2)
C5—C6	1.398 (3)	C14—C17	1.434 (3)
C5—H5	0.9300	C15—C16	1.382 (3)
C6—C7	1.376 (3)	C15—H15	0.9300
C6—C9A	1.452 (5)	C16—H16	0.9300
C6—C9B	1.603 (9)	C9B—C10B	1.310 (9)
C7—C8	1.350 (3)	C9B—H9B	0.9300
C7—H7	0.9300	C10B—H10B	0.9300
C8—H8	0.9300		
			
C3—N1—C1	120.45 (15)	C10A—C9A—H9A	119.5
C3—N1—C2	119.86 (16)	C6—C9A—H9A	119.5
C1—N1—C2	115.03 (16)	C10A—C9A—H10B	92.3
N1—C1—H1A	109.5	C6—C9A—H10B	146.5
N1—C1—H1B	109.5	C9A—C10A—C11	119.5 (6)
H1A—C1—H1B	109.5	C9A—C10A—H10A	120.3
N1—C1—H1C	109.5	C11—C10A—H10A	120.3
H1A—C1—H1C	109.5	C9A—C10A—H9B	98.2
H1B—C1—H1C	109.5	C11—C10A—H9B	141.5
N1—C2—H2A	109.5	C12—C11—C16	117.02 (19)
N1—C2—H2B	109.5	C12—C11—C10B	146.8 (4)
H2A—C2—H2B	109.5	C16—C11—C10B	96.2 (3)
N1—C2—H2C	109.5	C12—C11—C10A	110.3 (3)
H2A—C2—H2C	109.5	C16—C11—C10A	132.7 (3)
H2B—C2—H2C	109.5	C13—C12—C11	122.7 (2)
N1—C3—C8	121.29 (17)	C13—C12—H12	118.7
N1—C3—C4	122.25 (16)	C11—C12—H12	118.7
C8—C3—C4	116.45 (17)	C12—C13—C14	119.85 (18)
C5—C4—C3	120.92 (18)	C12—C13—H13	120.1
C5—C4—H4	119.5	C14—C13—H13	120.1
C3—C4—H4	119.5	C15—C14—C13	119.55 (17)
C4—C5—C6	122.2 (2)	C15—C14—C17	120.16 (18)
C4—C5—H5	118.9	C13—C14—C17	120.28 (17)
C6—C5—H5	118.9	C14—C15—C16	119.3 (2)
C7—C6—C5	115.18 (19)	C14—C15—H15	120.3
C7—C6—C9A	108.6 (3)	C16—C15—H15	120.3
C5—C6—C9A	136.2 (3)	C15—C16—C11	121.58 (19)
C7—C6—C9B	143.5 (3)	C15—C16—H16	119.2
C5—C6—C9B	101.4 (3)	C11—C16—H16	119.2
C8—C7—C6	123.7 (2)	N2—C17—C14	178.3 (2)
C8—C7—H7	118.2	C10B—C9B—C6	114.5 (9)
C6—C7—H7	118.2	C10B—C9B—H9B	122.7
C7—C8—C3	121.6 (2)	C6—C9B—H9B	122.8
C7—C8—H8	119.2	C9B—C10B—C11	114.2 (9)
C3—C8—H8	119.2	C9B—C10B—H10B	123.0
C10A—C9A—C6	121.0 (7)	C11—C10B—H10B	122.8
			
C1—N1—C3—C8	166.46 (18)	C9A—C10A—C11—C16	−4.6 (5)
C2—N1—C3—C8	11.9 (3)	C9A—C10A—C11—C10B	−2.9 (4)
C1—N1—C3—C4	−14.9 (3)	C16—C11—C12—C13	−0.5 (3)
C2—N1—C3—C4	−169.52 (18)	C10B—C11—C12—C13	−178.3 (4)
N1—C3—C4—C5	179.55 (15)	C10A—C11—C12—C13	179.7 (2)
C8—C3—C4—C5	−1.8 (3)	C11—C12—C13—C14	0.7 (3)
C3—C4—C5—C6	0.5 (3)	C12—C13—C14—C15	−0.7 (3)
C4—C5—C6—C7	1.3 (3)	C12—C13—C14—C17	−179.97 (16)
C4—C5—C6—C9A	−178.0 (2)	C13—C14—C15—C16	0.6 (3)
C4—C5—C6—C9B	−178.5 (2)	C17—C14—C15—C16	179.78 (16)
C5—C6—C7—C8	−2.1 (3)	C14—C15—C16—C11	−0.3 (3)
C9A—C6—C7—C8	177.5 (2)	C12—C11—C16—C15	0.3 (3)
C9B—C6—C7—C8	177.6 (4)	C10B—C11—C16—C15	179.1 (2)
C6—C7—C8—C3	0.9 (3)	C10A—C11—C16—C15	−179.9 (2)
N1—C3—C8—C7	179.79 (17)	C7—C6—C9B—C10B	−1.6 (7)
C4—C3—C8—C7	1.1 (3)	C5—C6—C9B—C10B	178.1 (4)
C7—C6—C9A—C10A	−176.8 (3)	C9A—C6—C9B—C10B	−1.3 (3)
C5—C6—C9A—C10A	2.5 (5)	C6—C9B—C10B—C11	179.1 (3)
C9B—C6—C9A—C10A	3.3 (4)	C12—C11—C10B—C9B	1.2 (7)
C6—C9A—C10A—C11	−177.4 (3)	C16—C11—C10B—C9B	−176.8 (4)
C9A—C10A—C11—C12	175.2 (3)	C10A—C11—C10B—C9B	4.4 (4)

### Hydrogen-bond geometry (Å, °)

**Table:**

*D*—H···*A*	*D*—H	H···*A*	*D*···*A*	*D*—H···*A*
C1—H1C···N2^i^	0.96	2.99	3.858 (3)	151
C2—H2C···N2^i^	0.96	2.80	3.646 (3)	147
C13—H13···N2^ii^	0.93	2.88	3.696 (3)	147

Symmetry codes: (i) *x*−3/2, −*y*+1/2, *z*+1/2; (ii) −*x*+2, −*y*+1, −*z*.

## References

[bb1] Allen, F. H. (2002). *Acta Cryst.* B**58**, 380–388. 10.1107/s0108768102003890 12037359

[bb2] Blanchard-Desce, M., Ledoux, I., Lehn, J. M., Malthete, J. & Zyss, J. (1988). *J. Chem. Soc. Chem. Commun.* pp. 737–739.

[bb3] Craig, N. C., Groner, P. & McKean, D. C. (2006). *J. Phys. Chem. A*, **110**, 7461–7469. 10.1021/jp060695b 16759136

[bb4] Daum, R., Hansson, T., Norenberg, R., Schwarzer, D. & Schroeder, J. (1995). *Chem. Phys. Lett.* **246**, 607–614.

[bb5] Etter, M. (1990). *Acc. Chem. Res.* **23**, 120–126.

[bb6] Farrugia, L. J. (1997). *J. Appl. Cryst.* **30**, 565.

[bb7] Kubicki, A. A. (2007). *Chem. Phys. Lett* **439**, 243–246.

[bb8] Lapouyade, R., Kuhn, A., Letard, J. F. & Retting, W. (1993). *Chem. Phys. Lett.* **208**, 48–58.

[bb9] Maryanoff, B. E. & Reitz, A. B. (1989). *J. Chem. Rev.* **89**, 863.

[bb10] Nardelli, M. (1995). *J. Appl. Cryst.* **28**, 659.

[bb11] Nonius (2000). *COLLECT* Nonius BV, Delft, The Netherlands.

[bb12] Otwinowski, Z. & Minor, W. (1997). *Methods in Enzymology*, Vol. 276, *Macromolecular Crystallography*, Part A, edited by C. W. Carter Jr & R. M. Sweet, pp. 307–326. New York: Academic Press.

[bb13] Papper, V., Pines, D., Likhtenshtein, G. & Pines, E. (1997). *J. Photochem. Photobiol. A*, **111**, 87–96.

[bb14] Sheldrick, G. M. (2008). *Acta Cryst.* A**64**, 112–122. 10.1107/S0108767307043930 18156677

